# Effectiveness and risk factors for virological outcome of darunavir-based therapy for treatment-experienced HIV-infected patients

**DOI:** 10.1186/s12981-015-0072-9

**Published:** 2015-09-24

**Authors:** José Antonio Mata-Marín, Gloria Huerta-García, Juan Carlos Domínguez-Hermosillo, Marcelino Chavez-García, Marco Isaac Banda-Lara, Nohemí Nuñez-Rodríguez, Javier Enrique Cruz-Herrera, Jorge Luis Sandoval-Ramírez, Ivan Martínez-Abarca, Alfredo Francisco Villagómez-Ruíz, Bulmaro Manjarrez-Tellez, Jesús Gaytán-Martínez

**Affiliations:** Infectious Diseases Department, National Medical Center “La Raza”, IMSS, Mexico Distrito Federal, Mexico; Pediatric Infectious Diseases Department, National Medical Center “Siglo XXI”, IMSS, Mexico Distrito Federal, Mexico; Infectious Diseases Department, Third Level Hospital No. 25, IMSS, Monterrey, NL Mexico; AIDS Clinic, General Hospital No 2, IMSS, San Luis Potosí, S.L.P. Mexico; AIDS Clinic, General Hospital No-24, IMSS, Mexico Distrito Federal, Mexico; AIDS Clinic, General Hospital No.72, IMSS, Mexico Distrito Federal, Mexico; AIDS Clinic, Regional Hospital No. 1, IMSS, Culiacán, Sinaloa Mexico; Seris y Jacarandas s/n, Colonia “La Raza”, Del Azcapotzalco, CP 02990 Ciudad de México D.F., Mexico

**Keywords:** Darunavir, HIV, Drug resistance, HIV-1 RNA, Virological outcome

## Abstract

**Objective:**

We evaluated the effectiveness of darunavir (DRV) treatment plus an optimized background regimen in 120 HIV-1 treatment-experienced patients.

**Design:**

Retrospective cohort, multicenter study.

**Methods:**

Adults >16 years with virological treatment failure starting therapy with a DRV-containing regimen were included. Effectiveness was evaluated as the percentage of patients with an undetectable HIV-1 RNA viral load (<50 and <200 copies/mL) after 48 weeks, and changes in CD4+ cell counts. We evaluated the risk factors associated with treatment failure.

**Results:**

Of the cohort, 83 % were men with a median age of 45 years (interquartile range, IQR 40–51). They had experienced treatment for a median of 13 years (IQR 9–17) with a median of six previous regimens (IQR 4–7), all using protease inhibitors. After treatment, 82 % (95 % confidence interval, CI 74–88 %) of patients had an HIV-1 RNA viral load <200 copies/mL and 69 % (95 % CI 60–76 %) had <50 copies/mL. The CD4+ cell count increased by 378 cells/μL (IQR 252–559; *P* < 0.001 vs. baseline). Risk factors associated with poor outcome were age >40 years [odds ratio, OR 0.15 (95 % CI 0.10–0.78); *P* = 0.015], use of raltegravir in the regimen [OR 0.37 (95 % CI 0.10–0.97); *P* = 0.046], and baseline CD4+ cell count <200 cells/μL [OR 2.79 (95 % CI 1.11–6.97); *P* = 0.028].

**Conclusion:**

In this Mexican cohort Darunavir was metabolically safe, well tolerated and achieved high rates of virological suppression in highly treatment-experienced patients infected with HIV-1.

## Background

Virological treatment failure occurs in a significant proportion of patients infected with HIV-1, and represents one of their most challenging health management issues. Cohort studies suggest that approximately 10 % of patients experience triple-class treatment failure, and these rates are increasing with time [1]. Protease inhibitors (PIs) have significantly improved the management of HIV infection. However, antiretroviral (ARV) drug resistance, including multi-drug and cross-resistance, compromises effective long-term therapy. PIs are capable of providing virological suppression, particularly in treatment-experienced patients infected with HIV [2]. Combined darunavir/ritonavir (DRV/r) treatment was approved by the U.S Food and Drug Administration in June 2006 for use in treatment-experienced patients [3, 4]. The clinical trials POWER 1 and POWER 2 exposed randomized highly treatment-experienced patients to DRV/r plus an optimized background regimen (OBR) or control PIs (CPI) plus an OBR. At 48 weeks, 61 % of patients in the DRV/r arm had a reduction of plasma viral load of around 1 log_(10)_ copies/mL versus 15 % in the CPI arm [5]. The results of the TITAN study, a large randomized phase III trial in lopinavir-naive, HIV-infected, treatment-experienced patients, showed that DRV/r was not inferior to lopinavir–ritonavir (LPV/r) therapy, as determined by the primary endpoint of <400 copies/mL of HIV RNA at week 48. Results of a secondary analysis showed that DRV/r was superior to LPV/r at this time point [6]. There are few studies in real-life situations. Thus, the Swiss Cohort Study reported that as a component of therapy for treatment-experienced patients, DRV/r could achieve a similar efficacy and tolerability in clinical practice to those seen in clinical trials. Clinicians should consider whether a patient has failed treatment with both lopinavir and saquinavir and the number of failed PI regimens before prescribing darunavir [7]. In Brazil, a cohort study was conducted with DRV/r plus an OBR and found that it was a highly effective salvage regimen under clinical routine conditions [8]. The aim of the present study was to evaluate the virological and immunological effectiveness of DRV plus an OBR in a Mexican cohort of highly treatment-experienced patients infected with HIV-1.

## Methods

### Design

We performed a retrospective study on a cohort of 120 treatment-experienced HIV-1-infected adults who started therapy with a DRV-containing regimen. The first endpoint to analyze was an HIV-1 RNA viral load of <50 copies/mL after the patients had completed 48 weeks of treatment. The secondary endpoint was an HIV-1 RNA viral load of <200 copies/mL and any increase in the CD4+ cell count. Finally, we evaluated the risk factors associated with treatment failure in these patients.

### Patients

Patients were recruited for HIV treatment from seven referral centers of four states in Mexico. Patients were >16 years of age with HIV-1 infection confirmed by enzyme-linked immunosorbent assay and western blotting, who had virological treatment failure from three classes of antiviral drugs, and had mutations detected. Patients had been treated previously with at least three classes of ARV drugs including nucleoside analog reverse-transcriptase inhibitors (NRTIs), non-nucleoside reverse-transcriptase inhibitors (NNRTIs), and protease inhibitors (PIs). Four patients had a history of integrase inhibitor use, with mutation resistance documented for each class. Seven patients had been treated unsuccessfully with enfuvirtide, but we did not have access to gp41 genotyping for them.

An individualized OBR was chosen for each patient. The regimen included three to four ARV agents, trying to use ≥2 fully active drugs according to HIV-1 resistance testing and previous ARV drug experience.

### Measurements

Clinical histories were recorded regarding ARV regimens, CD4+ cell counts, HIV-1 RNA viral load, and serum laboratory parameters at the beginning of the therapy with DRV (baseline) and at 48 weeks later. Once provided with each patient’s genotype, tropism-testing and previous regimens, an expert committee evaluated each case to decide the better option for a salvage regimen using DRV plus an OBR, considering the previous use of ARV regimens.

Mutations were assessed from plasma HIV-1 *pol* sequences using the Stanford HIV Drug Resistance Database (HIVdb; http://hivdb.stanford.edu). The presence of resistance was defined according to the Stanford HIVdb sensitivity score (SS) ranges as follows: 0–9 = susceptible; 10–14 = potential low-level resistance; 15–29 = low-level resistance; 30–59 = intermediate resistance; and ≥60 = high-level resistance.

The genotypic SS (GSS) was defined as the total number of drugs (excluding darunavir) in a participant’s OBR ARV regimen to which their HIV isolate had genotypic sensitivity, as deduced from gene sequence and mutation analyses. This was calculated based on the drug resistance scores extracted from the Stanford HIVdb. Each ARV drug was assigned a score according to the five-level Stanford HIVdb interpretation. The sum of the individual scores for specific drugs provided the total GSS of that treatment, where 0–9 = 1, 10–14 = 0.75, 15–29 = 0.5, 30–59 = 0.25 and >60 = 0. We classified the total GSS score in the following categories: 0–1, 1–2, or ≥2. The 0–1 group contains viral sequences almost entirely resistant to the drugs in the OBR regimen, and the ≥2 group contains viral sequences susceptible to more than two drugs given in the regimen [9].

The effectiveness of DRV treatment was evaluated based on the percentages of patients with an undetectable HIV-1 RNA viral load after 48 weeks of treatment. We also evaluated changes in CD4+ cell counts. We analyzed the resistance-associated mutations (RAMs) associated with DRV at baseline, OBR GSS and the DRV Stanford Score for potential risk factors of virological treatment failure. Evaluations of metabolic safety were based on changes in fasting lipid levels (total cholesterol and triglycerides), and creatinine from baseline to week 48.

### Statistical analysis

Baseline characteristics were summarized using medians and interquartile ranges (IQRs) for continuous variables, and proportions for categorical variables. Nonparametric paired tests were used to evaluate changes in CD4+ cell counts and HIV-1 RNA viral load. Descriptive statistics were used to evaluate changes in CD4+ cell counts and HIV-1 RNA viral load from baseline. For continuous variables, we calculated medians with IQRs. For categorical variables, we calculated the number of values in each category and the percentages of the values with regard to the number of patients. Explorative statistical methods were used regarding the efficacy endpoints and changes in safety-relevant laboratory parameters. Significant changes from baseline were tested using the Wilcoxon signed-rank test. We calculated the 95 % confidence interval (CI) for appropriate results.

Baseline differences between patients who reached or did not reach a viral load of <50 copies/mL at week 48 were tested using bivariate analysis, which included crude odds ratios (ORs), Fisher’s exact and Chi squared tests. Independent risk factors associated with virological response at week 48 were identified in the multivariate logistic regression analysis that included variables from bivariate analysis. All analyses were carried out using SPSS software (IBM Corp. Released 2010. IBM SPSS Statistics for Windows, Version 19.0. Armonk, NY, USA: IBM Corp.).

## Results

A total of 136 multidrug-experienced patients who started a DRV/r-based salvage therapy between 2009 and 2013 were identified. Ten were excluded because they had incomplete data in the files. Four patients experienced rashes at the beginning of the regimen and they were not considered for the analysis; two patients changed their institution and we did not have any follow-up data for them. Thus, we finally included 120 patients who were followed through the 48-week retrospective analyses. The median age of the overall cohort at DRV initiation was 45 years (IQR 40–51) and 83 % were men. Center of Disease Control Class C AIDS was found in 68 % of patients and the median number of previous ARV treatments was six (IQR 4–7). All patients had experience of prior PI use, most with indinavir, saquinavir/ritonavir and lopinavir/ritonavir (Table 1).

**Table 1:** Baseline patient characteristics and optimized background

Characteristics	Values^a^
Age, years	45 (40–51)
Male gender, n (%)	102 (85 %)
Number of previous regimens	6 (4–7)
Years of experienced treatment	13 (9–17)
Baseline HIV-1 plasma viral load, log_10_ copies/mL	4.35 (3.55–4.66)
Baseline HIV-1 RNA >100,000 copies/mL, n (%)	24 (20 %)
Baseline CD4+ cell counts (cells/μL)	245 (129–400)
Baseline CD4+ cells count <200 cells/μL, n (%)	45 (37.5)
Number of mutations to DRV	1 (0–2)
Number of mutations to DRV ≥2	47 (39 %)
Number of PI RAMs, non-DRV RAMs	4 (3–6)
GSS^b^ for DRV	0.75 (0.5–1.0)
GSS^b^ for OBR	1.5 (1.2–2.0)
TDF in regimen, n (%)	77 (64.2 %)
MVC in regimen, n (%)	13 (10.8 %)
ENF in regimen, n (%)	13 (10.8 %)
ETV in regimen, n (%)	33 (27.5 %)
RAL in regimen, n (%)	106 (88.3 %)
Stanford score for TDF	35 (20–55)
Stanford score for ETV	10 (0–30)
Stanford score for DRV	12.5 (0–20)
Glucose (mg/dL)	85 (79–91)
Creatinine (mg/dL)	0.86 (0.70–1.0)
ALT (IU/dL)	30 (19–48)
Total cholesterol (mg/dL)	167 (136–195)
Triglycerides (mg/dL)	186 (135–260)

*ALT* alanine transaminase, *DRV* darunavir, *ENF* enfuvirtide, *ETV* etravirine, *GSS* genotypic susceptibility score, *MVC* maraviroc, *PI* protease inhibitor, *RAL* raltegravir, *RAM* resistance-associated mutation, *TDF* tenofovir

^a^Values are medians with (interquartile ranges), unless indicated otherwise

^b^Genotypic score according to the Stanford HIVdb

The primary endpoint was achieved in 69.2 % of patients (95 % CI 60–76 %) and the secondary endpoint in 82.5 % (95 % CI 74–88 %). At baseline, the median HIV-1-RNA viral load was 22,600 copies/mL (4.35 log_(10)_) with an IQR of 3590–75,797 copies/mL (3.5–4.8 log_(10)_). After 48 weeks of treatment, 69 % of patients (*n* = 83) had an HIV-1 RNA viral load of <50 copies/mL and 82 % (*n* = 98) had <200 copies/mL. The median was <50 copies/mL (<1.6 log_(10)_) with an IQR of <50–<85 copies/mL (<1.6–<1.9 log_(10)_) in both cases (*P* < 0.001 compared with baseline). At baseline, the median and IQR values for the CD4+ cell counts were 245 cells/μL (129–400 cells/μL), whereas at weeks 24 and 48 the values were 311 cells/μL (239–522 cells/μL) and 378 cells/μL (252–559 cells/μL), respectively. These increases were both statistically significant (*P* < 0.001).

The median GSS of the OBR for all patients was 1.5 (IQR 1.2–2.0). Forty-four (36.6 %) patients had a GSS ≥2 (Table 1). When we analyzed OBRs according to the Stanford HIVdb, we did not find significant differences between the GSS of those OBRs with treatment failure and those with <50 copies/mL. The median GSS for DRV according to the Stanford HIVdb was 12.5 (IQR 0–20).

The most frequent RAMs for DRV were L33F (35 %), I84V (33 %), L10F (18 %), V32I (7 %) and V11I (5 %). We were not able to find any relationship between the number of DRV mutations and virological failure (Table 2).

**Table 2:** Darunavir resistance mutations (*n* = 120)

Mutation	n (%)
L33F	42 (35 %)
I84V	40 (33 %)
L10F	22 (18 %)
I47V	12 (10 %)
V32I	9 (7 %)
V11I	7 (5 %)
I50V	5 (4 %)
T74P	5 (4 %)
L76V	5 (4 %)
L89V	5 (4 %)
I54M	3 (2 %)
I54L	2 (1 %)

The most common regimens associated with DRV/r were: tenofovir disoproxil fumarate (TDF) + raltegravir (RAL) (40 %), etravirine (ETV) + RAL (14.2 %) and TDF + ETV + RAL (6.5 %); the remaining regimens were used in 5 % or fewer of the patients. When we evaluated the risk factors for virological failure, we found that the association of DRV/r with some drugs increased the risk of failure but some drugs decreased it (Tables 3, 4).

**Table 3:** Bivariate and multivariate analysis of risk factors associated with a virological outcome of HIV-1 RNA <50 copies/mL at week 48 of antiretroviral treatment

Risk factor	Bivariate			Multivariate		
	OR unadjusted	95 % CI	P	OR adjusted	95 % CI	P
Male gender	1.14	0.39–3.32	0.803			
Age >40 years	0.35	0.14–0.88	0.023	0.28	0.10–0.78	0.015
Duration of ART >14 years	0.69	0.31–1.50	0.350			
Number of previous regimens ≥6	0.97	0.36–2.62	0.963			
Baseline HIV-1 RNA >100,000 copies/mL	2.84	1.13–7–13	0.023	2.10	0.74–5.90	0.158
Baseline CD4+ cell count <200 cells/μL	2.72	1.22–6–06	0.012	2.79	1.11–6.97	0.028
Number of mutations to DRV ≥2	0.922	0.41–2.04	0.842			
GSS in OBR <1 vs. ≥1	1.028	0.97–1.08	0.308			
GSS in OBR ≤1 vs. >1	1.820	0.56–5.91	0.235			
GSS in OBR <2 vs. ≥2	0.863	0.38–1.93	0.721			
TDF in regimen	1.48	0.64–3.40	0.236			
MVC in regimen	0.64	0.16–2.49	0.521			
ENF in regimen	2.99	0.93–9.64	0.057			
ETV in regimen	0.790	0.32–1.92	0.603			
RAL in regimen	0.28	0.09–0.88	0.023	0.37	0.10–0.97	0.046

*DRV* darunavir, *ENF* enfuvirtide, *ETV* etravirine, *GSS* genotypic susceptibility score, *MVC* maraviroc, *PI* protease inhibitor, *RAL* raltegravir, *RAM* resistance-associated mutation, *TDF* tenofovir

**Table 4:** Bivariate and multivariate analysis of factors associated with an outcome of HIV-1 RNA <200 copies/mL at week 48 of antiretroviral treatment

Risk factor	OR unadjusted	95 % CI	P	OR adjusted	95 % CI	P
Male gender	2.06	0.64–6.60	0.179			
Age >40 years	0.23	0.08–0.66	0.004	0.15	0.04–0.52	0.003
Duration of ART >14 years	0.55	0.21–1.43	0.218			
Number of previous regimens ≥6	1.40	0.45–4.33	0.372			
Baseline HIV-1 RNA >100,000 copies/mL	3.19	1.13–8.94	0.022	2.83	0.80–9.94	0.103
Baseline CD4+ cells count <200 cells/μL	2.66	1.02–6.96	0.041	2.65	0.80–8.76	0.109
Number of mutations to DRV ≥2	1.20	0.46–3.12	0.703			
GSS in OBR <1 vs. ≥1	1.05	0.95–1.15	0.175			
GSS in OBR ≤1 vs. >1	0.759	0.22–2.57	0.435			
GSS in OBR <2 vs. ≥2	0.615	0.22–1.72	0.352			
TDF in regimen	4.06	1.12–14.7	0.018	4.16	1.02–16.8	0.046
MVC in regimen	1.48	0.37–5.93	0.575			
ENF in regimen	1.48	0.37–5.93	0.406			
ETV in regimen	0.56	0.17–1.83	0.340			
RAL in regimen	0.22	0.06–0.72	0.008	0.32	0.08–1.24	0.101

*DRV* darunavir, *ENF* enfuvirtide, *ETV* etravirine, *GSS* genotypic susceptibility score, *MVC* maraviroc, *PI* protease inhibitor, *RAL* raltegravir, *RAM* resistance-associated mutation, *TDF* tenofovir

After applying a logistic regression model the following factors remained significant for the outcome of HIV-1 RNA <50 copies/mL: age >40 years [OR 0.15 (95 % CI 0.10–0.78); *P* = 0.015], the use of RAL in a regimen [OR 0.37 (95 % CI 0.10–0.97); *P* = 0.046], and CD4+ cell count <200 cells/μL [OR 2.79 (95 % CI 1.11–6.97); *P* = 0.028].

For assessing metabolic safety, fasting lipid profiles of total cholesterol (TC), and triglycerides (TG) were measured and are shown below as medians and IQRs. TC showed a significant increase (*P* = 0.003) from a baseline of 167 mg/dL (136–195 mg/dL) to 174 mg/dL (156–214 mg/dL) at week 24, and it remained significant at 48 weeks at 185 mg/dL (150–214 mg/dL; *P* < 0.001). TG showed no significant increase from a baseline of 186 mg/dL (135–260 mg/dL) to 197 mg/dL (148–271 mg/dL) at week 24, and it remained not significant at 48 weeks at 220 mg/dL (162–305 mg/dL) (Table 5).

**Table 5:** Endpoints after 24 and 48 weeks of treatment

Outcomes	Median (IQR)			*P* 24/48 weeks
	Baseline	Week 24	Week 48	
CD4+ cell count	245 (129–400)	311 (239–522)	378 (252–559)	<0.001*
HIV-1 RNA viral load	22,600 (3590–75,797)	<50 (<50–82)	<50 (<50–85)	<0.001*
Cholesterol (mg/dL)	167 (364–195)	174 (156–214)	185 (150–214)	0.004/<0.001
Triglycerides (mg/dL)	186 (135–260)	197 (148–271)	220 (162–305)	0.465/0.076
Creatinine (mg/dL)	0.86 (0.70–1.0)	0.9 (0.8–1.07)	0.9 (0.8–1.05)	<0.001*

* At both 24 and 48 weeks

## Discussion

This study has shown the feasibility of achieving good virological and immunological responses with DRV/r plus an OBR among highly ARV-experienced HIV-infected patients under routine clinical care, based on a multicenter cohort in Mexico. This particular setting allowed the evaluation of DRV/r under ‘real-life’ conditions. We found that a CD4+ cell count <200 cells/μL was associated with treatment failure, and the use of RAL in the regimen and age >40 years were associated with treatment success by achieving an HIV-1 RNA viral load of <50 copies/mL. In contrast, age >40 years was significantly associated with success, and TDF in the regimen was associated with failure leading to an HIV-1 RNA viral load of <200 copies/mL. Regarding metabolic safety, we had an increase in the TC levels after starting the new regimen but not in TGs.

We observed that 69 % of patients reached a viral load of <50 copies/mL at week 48. This outcome was better than some other randomized clinical trials [5], but was similar to the TITAN6 and ODIN studies at 48 weeks [10] and some observational studies [11, 12].

The concurrent use of RAL has been associated with a favorable treatment outcome. Some trials have found a synergistic effect such as the BENCHMRK clinical trial, where the coadministration of RAL and DRV/r improved the outcomes [13]. In that trial, HIV-1 RNA levels of <50 copies/mL were achieved at 48 weeks in 47 % of recipients of DRV/r plus an OBR, compared with 69 % of patients who received RAL and DRV/r plus an OBR; however, when we evaluated other drugs such as enfuvirtide (ENF) and maraviroc (MVC), we did not find the same result. Another observational study found that the concomitant use of RAL was strongly associated with treatment success [11].

Given that an OBR that included TDF was found to increase the risk of treatment failure, we found that an intermediate resistance for TDF in the SS (>35) was also associated with virological failure [OR 3.6 (95 % CI 1.17–11.00); *P* = 0.017] and 45 of the patients were using TDF with a high level of resistance. M41L was the most frequent mutation in our patients with intermediate or high level resistance, and this was associated with an increase in the risk of virological treatment failure in another study [14].

We found that age <40 years was associated with a better response that was attributed to a better adherence in this age group. A meta-analysis showed that older age reduces the risk for nonadherence by 25 % in short-term (relative risk, RR 0.75; 95 % CI 0.64–0.87) and 35 % in longer-term assessments (RR 0.65, 95 % CI 0.50–0.85) [15].

This is the first multicenter cohort in Mexico that has evaluated the effectiveness of DRV in highly treatment-experienced patients; a strength of this study was that we could evaluate risk factors for virological treatment failure. By contrast, one of the limitations of this study is that it was a retrospective method to study previously enrolled patients. In addition, we were not able to establish adverse events associated with DRV. Another limitation of this study is that we could not assess adherence to ARV drugs in our population.

## Conclusions

These results suggest that the use of DRV/r-based regimens for salvage therapy is an effective strategy in the clinical care setting of a developing country. DRV/r in combination with RAL increased the overall success as a regimen for salvage therapy in these highly ARV treatment-experienced HIV-infected patients. Finally, the use of TDF appears to be a good option except for those patients with an SS >35.

## References

[1] Mocroft A, Ledergerber B, Viard JP, Staszewski S, Murphy M, Chiesi A (2004). Time to virological failure of 3 classes of antiretrovirals after initiation of highly active antiretroviral therapy: results from the EuroSIDA study group. J Infect Dis.

[2] Katlama C, Esposito R, Gatell JM, Goffard JC, Grinsztejn B, Pozniak A (2007). Efficacy and safety of TMC114/ritonavir in treatment-experienced HIV patients: 24-week results of POWER 1. AIDS.

[3] Renaud-Thery F, Nguimfack BD, Vitoria M, Lee E, Graaff P, Samb B (2007). Use of antiretroviral therapy in resource-limited countries in 2006: distribution and uptake of first- and second-line regimens. AIDS.

[4] Wilson LE, Gallant JE (2009). The management of treatment-experienced HIV-infected patients: new drugs and drug combinations. Clin Infect Dis.

[5] Clotet B, Bellos N, Molina JM, Cooper D, Goffard JC, Lazzarin A (2007). Efficacy and safety of darunavir–ritonavir at week 48 in treatment-experienced patients with HIV-1 infection in POWER 1 and 2: a pooled subgroup analysis of data from two randomised trials. Lancet.

[6] Madruga JV, Berger D, McMurchie M, Suter F, Banhegyi D, Ruxrungtham K (2007). Efficacy and safety of darunavir–ritonavir compared with that of lopinavir–ritonavir at 48 weeks in treatment-experienced, HIV-infected patients in TITAN: a randomised controlled phase III trial. Lancet.

[7] Young J, Scherrer AU, Günthard HF, Opravil M, Yerly S, Böni J (2011). Efficacy, tolerability and risk factors for virological failure of darunavir-based therapy for treatment-experienced HIV-infected patients: the Swiss HIV Cohort Study. HIV Med.

[8] Vidal JE, Song AT, Matos ML, Bartmann D, Anjos GD, Miranda ÉJ (2013). High rate of virologic suppression with darunavir/ritonavir plus optimized background therapy among highly antiretroviral-experienced HIV-infected patients: results of a prospective cohort study in São Paulo, Brazil. Braz J Infect Dis.

[9] Frentz D, Boucher CA, Assel M, De Luca A, Fabbiani M, Incardona F (2010). Comparison of HIV-1 genotypic resistance test interpretation systems in predicting virological outcomes over time. PLoS One.

[10] Cahn P, Fourie J, Grinsztejn B, Hodder S, Molina JM, Ruxrungtham K (2011). Week 48 analysis of once-daily vs. twice-daily darunavir/ritonavir in treatment-experienced HIV-1-infected patients. AIDS.

[11] Biscione FM, Westin MR, Ribeiro KM, Estevam DL, Cardoso SW, Tenore SB (2014). Virologic and immunologic effectiveness at 48 weeks of darunavir–ritonavir-based regimens in treatment-experienced persons living with HIV-1 infection in clinical practice: a multicenter Brazilian cohort. J Int Assoc Provid AIDS Care.

[12] Delaugerre C, Buyck JF, Peytavin G, Viard JP, Chaix ML, Zucman D (2010). Factors predictive of successful darunavir/ritonavir-based therapy in highly antiretroviral-experienced HIV-1-infected patients (the DARWEST study). J Clin Virol.

[13] Cooper DA, Steigbigel RT, Gatell JM, Rockstroh JK, Katlama C, Yeni P (2008). Subgroup and resistance analyses of raltegravir for resistant HIV-1 infection. N Engl J Med.

[14] Di Giambenedetto S, Torti C, Prosperi M, Manca N, Lapadula G, Paraninfo G (2009). Effectiveness of antiretroviral regimens containing abacavir with tenofovir in treatment-experienced patients: predictors of virological response and drug resistance evolution in a multi-cohort study. Infection.

[15] Ghidei L, Simone MJ, Salow MJ, Zimmerman KM, Paquin AM, Skarf LM (2013). Aging, antiretrovirals, and adherence: a meta analysis of adherence among older HIV-infected individuals. Drugs Aging.

